# Supplementary material for: Income-based differences in healthcare utilization in relation to mortality in the Swedish population between 2004–2017: A nationwide register study
Source: PLoS Med. 2023 Nov 16;20(11):e1004230. doi: 10.1371/journal.pmed.1004230 (PMC10653442; doi:10.1371/journal.pmed.1004230)
Supplement: S1 Appendix — (DOCX) [file pmed.1004230.s002.docx]

S1 Appendix – supplemental method

Income measure

The income data were provided by Statistics Sweden and is deemed to be a reliable indicator and is commonly used in research. However it does not capture wealth [1].

We used the variable **DispInkKE04** provided by Statistics Sweden and described as [“..*a recommended variable often used in the research world and also in the statistics produced within the EU and other national statistical agencies. … the disposable income per consumption unit is obtained by dividing the sum of all disposable income of the members included in the family by the family's total consumption weight. Negative values can occur.*

*Example: A family in 1993, consisting of two adults, one child 0-3 years, and one child 4-10 years. The family's total consumption weight = 1.92 (two adults) + 0.56 (child 0-3 years) + 0.66 (child 4-10 years) = 3.14. The family's disposable income = 300,000 kr. The individual's (adult) disposable income = 300,000 / 3.14 = 95,541 kr.”]* (p. 319, translated from Swedish by the authors.)

Statistical analyses

#### Statistical analysis of (adjusted) Odds Ratios

Statistical analysis of cross sectional (yearly) odds ratios comparing the lowest income group (Q1) with the highest (Q5) for each disease group, and for each type of data (primary care, outpatient, inpatient and mortality). The logistic regression for our main analysis, Model 1, was specified as follows:

$$logit\left( P\left( Y=1 \right) \right)=\beta_{0}+\beta_{1-4}* IncomeRank+ \beta_{5}*Sex+\beta_{6-7}*birth country+\beta_{8}*age +\beta_{9}*{age}^{2} + \beta_{10-12}*civil status$$

where P(Y=1) represents the probability of having the outcome of interest, and $\beta_{0-12}$ are the coefficients associated with the intercept and independent variables. The effects of interest were IncomeRank, a categorical variable of 4 income groups, where the highest (Q5) where modelled as an implicit reference, and the reported adjusted OR were obtained by:

OR = exp($\boldsymbol{\beta}_{\boldsymbol{1}}$)

Furthermore, in the model we adjusted for sex (males or females), age (in years), age squared, country of birth (defined either as Sweden, Europe except Sweden, and outside Europe), and civil status (married or other).

In the unadjusted model, Model 2, only income rank was included.

$$logit\left( P\left( Y=1 \right) \right)=\beta_{0}+\beta_{1-4}* IncomeRank$$

In a sensitivity analysis, Model 3, we included the same regressors as for the main model (Model 1) and additional categorical regressors controlling for the 21 counties.

$$logit\left( P\left( Y=1 \right) \right)=\beta_{0}+\beta_{1-4}* IncomeRank+ \beta_{5}*Sex+\beta_{6-7}*birth country+\beta_{8}*age +\beta_{9}*{age}^{2} + \beta_{10-12}*civil status+ \beta_{13-33}*county$$

To quantify linear trends in the time series of OR, we first log-transformed the ORs to obtain log-odds scales. To account for uncertainty in the log-transformed ORs, 1000 new timeseries were simulated for each original OR time series. For each simulated time series, new parameter estimates pertaining to time were obtained by:

$$\boldsymbol{log}\left( \boldsymbol{OR}\boldsymbol{\_}\boldsymbol{simulated} \right)\mathbf{=}\boldsymbol{\beta}_{\mathbf{0}}\mathbf{+}\boldsymbol{\beta}_{\mathbf{1}}\mathbf{*}\boldsymbol{years}$$

Based on the distribution of the 1000 estimated $\boldsymbol{\beta}_{\mathbf{1}}\boldsymbol{s}$ we calculated the 95% confidence intervals.

#### Statistical analysis of (adjusted) Rate Ratios

Similar to the logistic regression, three models were tested, and the same sets of covariates were used. Thus, for the main model (Model 1) we calculated the adjusted Rate Ratios (RR) of number of health care encounters using negative binominal regressions:

$$logit\left( E(Y \right)=\beta_{0}+\beta_{1-4}* IncomeRank+ \beta_{5}*Sex+\beta_{6-7}*birth country+\beta_{8}*age +\beta_{9}*{age}^{2} + \beta_{10-12}*civil status*civil status$$

where E(Y) is the expected value of the outcome variable (i.e., number of health care encounters). The parameter estimates $\boldsymbol{\beta}_{\mathbf{0-12}}$ and pertaining regressors were analogous to the ones described for Model 1 the Logistic Regression above. Thus, the reported adjusted Rate Ratio were obtained by

RR = exp($\boldsymbol{\beta}_{\boldsymbol{1}}$).

As for the logistic regression, RR were also calculated without adjustments (Model 2), and by adjusting for the same set of covariates as used in the main analysis (Model 1) but also including counties (Model 3). Linear time trends in log-transformed RR (2008 – 2017) were calculated in a similar fashion as time trends of log-transformed OR.

References

1. Statistics Sweden. Longitudinell integrationsdatabas för Sjukförsäkrings-och Arbetsmarknadsstudier (LISA) 1990–2013. Arbetsmarknad och Utbildning Bakgrundsfakta 2016: 1. 2016
