# Supplementary material for: Income-based differences in healthcare utilization in relation to mortality in the Swedish population between 2004–2017: A nationwide register study
Source: PLoS Med. 2023 Nov 16;20(11):e1004230. doi: 10.1371/journal.pmed.1004230 (PMC10653442; doi:10.1371/journal.pmed.1004230)
Supplement: S1 Table — Primary care data did not necessarily constitute the basis for disbursements, and the coverage rates could not be well verified and likely improved with time. (DOCX) [file pmed.1004230.s003.docx]

| **County** | **Inclusive start year** | **Contact information to data providers** |
| --- | --- | --- |
| 01 - Stockholm | 2004 | https://www.regionstockholm.se/om-regionstockholm/forskning-och-innovation/centrum-for-halsodata/fragor-och-svar-for-forskare-om-halsodatabaser/ |
| 03 - Uppsala | 2010 | https://regionuppsala.se/samverkanswebben/it-service-och-fastighet/it-system/sas-viya/bestallning-av-data-for-forskning/ |
| 05 - Östergötland | 1999 | https://www.regionostergotland.se/ro/det-har-gor-vi/forskning/for-dig-som-forskar/forskarservice-och-infrastruktur/ansokan-om-registeruttag-fran-vardregister |
| 06 - Jönköping | 2012 | fouenheten@rjl.se |
| 08 - Kalmar | 2011 | primarvardsforvaltningen@ltkalmar.se |
| 09 - Gotland | 2013 | statistikehalsamit@gotland.se, |
| 10 - Blekinge | 2010 | region@regionblekinge.se |
| 12 - Skåne | 2004 | https://vardgivare.skane.se/kompetens-utveckling/forskning-inom-region-skane/utlamnande-av-patientdata-samradkvb/ |
| 14 - Västra Götaland | 2000 | https://www.vgregion.se/statistik-analysportalen/bestalla-statistik-och-data/ |
| 17 - Värmland | 2014 | https://www.regionvarmland.se/ |
| 18 - Örebro | 2002 | https://www.regionorebrolan.se/sv/forskning/kontakt-och-organisation/Forskning--och-utbildningsledning/ |
| 19 - Västmanland | 2017 | forskning@regionvastmanland.se |
| 20 - Dalarna | 2005 | https://www.regiondalarna.se/plus/forskning/personuppgifter-till-forskning/ |
| 21 - Gävleborg | 2010 | https://www.regiongavleborg.se/samverkanswebben/halsa-vard-tandvard/samverkan-och-avtal/halsovalet/Kontakt/ |
| 22 - Västernorrland | 2011 | https://www.rvn.se/sv/Om-regionen/regionens-organisation/patientsakerhet-utveckling-och-forskning/Forskning-och-utbildning/fouu-och-folkhalsa/Personal/ |
| 23 - Jämtland | 2010 | Beslutsstod@regionjh.se |
| 25 - Norrbotten | 2012 | forskning@norrbotten.se |
