# Supplementary material for: Income-based differences in healthcare utilization in relation to mortality in the Swedish population between 2004–2017: A nationwide register study
Source: PLoS Med. 2023 Nov 16;20(11):e1004230. doi: 10.1371/journal.pmed.1004230 (PMC10653442; doi:10.1371/journal.pmed.1004230)
Supplement: S2 Table — (DOCX) [file pmed.1004230.s004.docx]

| ***Disease group*** | ***ICD-10 codes*** |
| --- | --- |
| All-disease | * (all ICD codes) |
| Cardovascular diseases | B33.2-B33.24, D86.85, G45-G46.8, I01-I01.9, I02.0, I05-I09.9, I11-I11.2, I11.9, I20-I21.6, I21.9-I27.0, I27.2-I28.9, I30-I38.0, I39-I41.8, I42-I43.8, I44-I44.8, I45-I52.8, I60-I64, I64.1, I65-I83.93, I86-I89.0, I89.9, I95.0-I95.1, I98, I98.8-I99.9, K75.1, R00-R01.2, Z01.3-Z01.31, Z03.4-Z03.5, Z13.6, Z52.7, Z82.3-Z82.49, Z86.7-Z86.79, Z94.1-Z94.3, Z95-Z95.9 |
| Neoplasms | C00-C07, C08-C19.0, C20, C21-C21.8, C22-C22.4, C22.7-C23, C24-C26.1, C26.8-C26.9, C30-C30.1, C31-C33, C34-C34.92, C37-C37.0, C38-C39.9, C40-C41.4, C41.8-C41.9, C43-C45.2, C45.7, C45.9, C47-C4A, C50-C50.629, C50.8-C52, C53-C54.3, C54.8-C56.2, C56.9-C58.0, C60-C64.2, C64.9-C69.92, C70-C70.1, C70.9-C73, C74-C75.5, C75.8-C79.9, C80-C81.49, C81.7-C81.79, C81.9-C85.29, C85.7-C86.6, C88-C90.32, C91-C93.7, C93.9-C95.2, C95.7-C97.9, D00-D24.9, D26.0-D39.9, D4-D49.9, E34.0, K51.4-K51.419, K62.0-K62.3, K63.5, N60-N60.99, N84.0-N84.1, N87-N87.9, Z03.1, Z08-Z09.9, Z12-Z12.9, Z80-Z80.9, Z85-Z85.9, Z86.0-Z86.03 |
| Neurological disorders | F00-F02.0, F02.2-F02.3, F02.8-F03.91, F06.2, G10-G10.0, G11-G13.8, G20-G21, G21.2-G24, G24.1-G25.0, G25.2-G25.3, G25.5, G25.8-G26.0, G30-G31.1, G31.8-G32.89, G35-G35.0, G36-G37.9, G40-G41.9, G43-G44.89, G50-G54.1, G54.5-G62, G62.2-G65.2, G70-G71.19, G71.3-G72, G72.1-G73.7, G80-G83.9, G89-G93.6, G93.8-G95.29, G95.8-G96, G96.1, G96.12-G96.9, G98-G99.8, M33-M33.99, M60-M60.19, M60.8-M60.9, M79.7, R25-R27.9, R29-R29.91, R41-R42.0, R56-R56.9, R90-R90.89, Z03.3, Z13.85, Z13.858, Z82.0, Z86.6-Z86.69 |
| Chronic respiratory diseases | 189D86-D86.2, D86.9, G47.3-G47.39, J30-J35.9, J37-J39.9, J41-J42.4, J43-J46.0, J47-J47.9, J60-J68.9, J70.8-J70.9, J80-J80.9, J82, J84-J84.9, J90-J90.0, J91, J91.8-J93.12, J93.8-J94.9, J96-J96.92, J98-J99.8, R05.0-R06.9, R09-R09.89, R84-R84.9, R91-R91.8, Z82.5 |
| Diabetes and kidney diseases | D63.1, E08-E08.9, E10-E14.9, I12-I13.9, N00-N08.8, N15.0, N17-N19, Q60-Q63.2, Q63.8-Q63.9, Q64.2-Q64.9, R73-R73.9, Z13.1, Z49-Z49.32, Z52.4, Z83.3, Z99.2 |
