# Supplementary figures and images for: Income-based differences in healthcare utilization in relation to mortality in the Swedish population between 2004–2017: A nationwide register study
Source: PLoS Med. 2023 Nov 16;20(11):e1004230. doi: 10.1371/journal.pmed.1004230 (PMC10653442; doi:10.1371/journal.pmed.1004230)

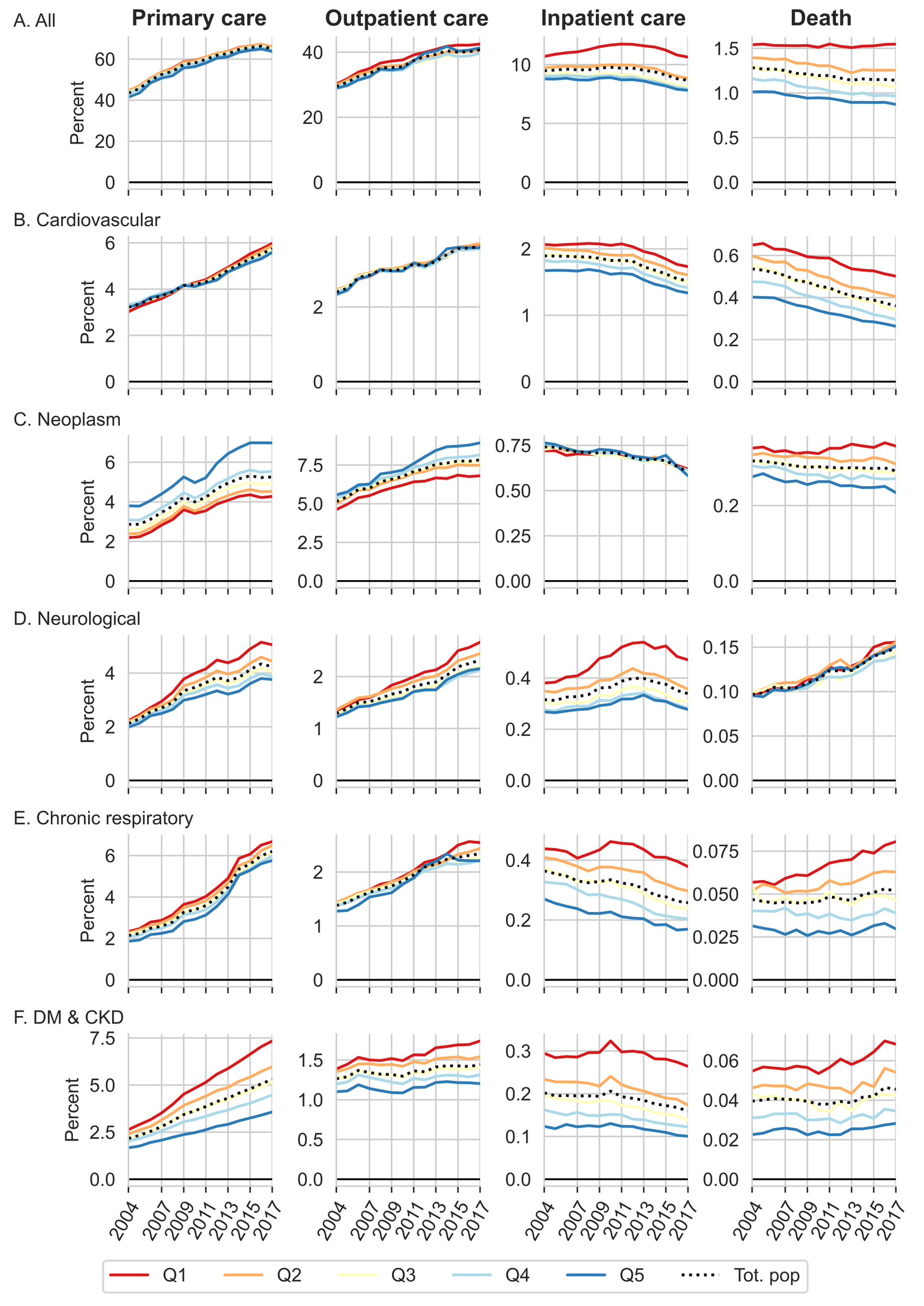

Supplement: S1 Fig — Q1, lowest income quantile; Q5, highest income quantile. All, all-disease; DM & CKD, diabetes and chronic kidney diseases. (TIF) [file pmed.1004230.s008.tif]

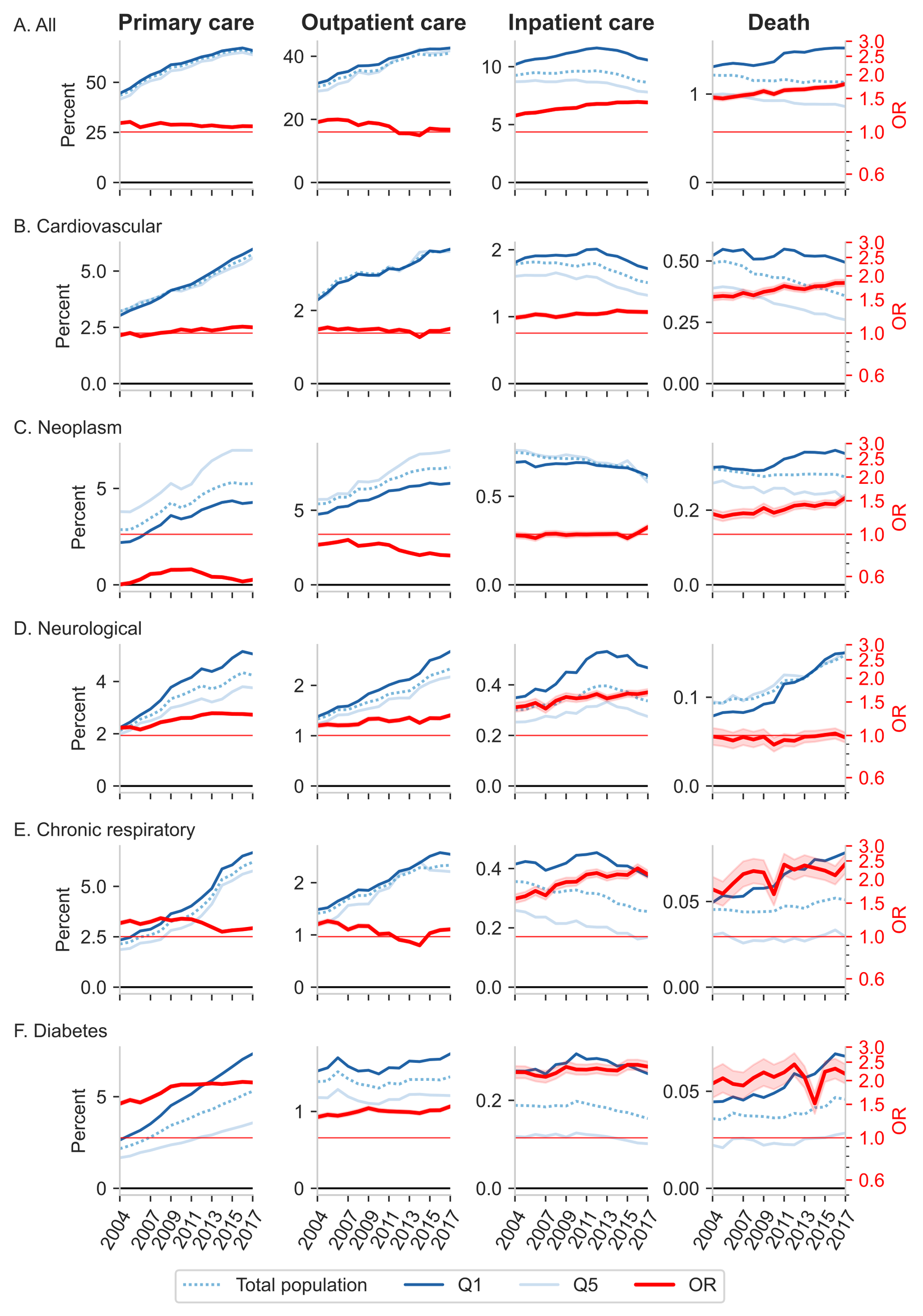

Supplement: S2 Fig — This sensitivity analysis confirms the robustness of the results shown in Fig 2 in relation to variations in the study sample. HCU (yearly rates of unique individuals) and mortality rates, by income group and for the total study population, are shown in blue colors. Rates are presented in percentages for display purposes. Adjusted ORs (95% CI) comparing Q1 with Q5 are in red, and shaded areas denote 95% CI. ORs are adjusted for sex, age, age squared, country of birth, and civil status. Red horizontal grid lines denote OR = 1, and the black grid lines denote the zero levels of rates. All, any ICD-10 code; CVD, cardiovascular diseases; Neuro, neurological disorders; Chro. Resp., chronic respiratory diseases; DM & CKD, diabetes and kidney diseases; HCU, healthcare utilization; OR, odds ratio. (TIF) [file pmed.1004230.s009.tif]
