# Supplementary material for: Income-based differences in healthcare utilization in relation to mortality in the Swedish population between 2004–2017: A nationwide register study
Source: PLoS Med. 2023 Nov 16;20(11):e1004230. doi: 10.1371/journal.pmed.1004230 (PMC10653442; doi:10.1371/journal.pmed.1004230)
Supplement: Attachment — Submitted filename: Response_to_reviewers.docx [file pmed.1004230.s010.docx]

Dear Editor and reviewers,

We are sincerely grateful for your thorough evaluation of our manuscript and your valuable feedback. We have carefully considered all your comments and am providing a point-by-point response in (**bold)** to each of them below:

-----------------------------------------------------------
Requests from the editors:

GENERAL
Please respond to all editor and reviewer comments detailed below in full.

Please ensure that the study is reported according to the STROBE guideline, and include the completed STROBE checklist as Supporting Information. Please add the following statement, or similar, to the Methods: "This study is reported as per the Strengthening the Reporting of Observational Studies in Epidemiology (STROBE) guideline (S1 Checklist)."

The STROBE guideline can be found here: http://www.equator-network.org/reporting-guidelines/strobe/
When completing the checklist, please use section and paragraph numbers, rather than page (and/or line) numbers as these often change in the event of publication.

DATA AVAILABILITY STATEMENT
Please include an appropriate email or web address for the Swedish National Board of Health and Wellfare and Statistics Sweden.

**- Web address for the Swedish National Board of Health and Welfare and Statistics Sweden are now stated. In addition, S1 Table is now updated with contact information to the providers of primary care data.**

COMMENTS FROM THE ACADEMIC EDITOR
This is an interesting paper and I agree with a major revision. I agree that adjusting for baseline health status is important. Reviewer 1 in particular has very helpful input. One of my concerns is the lack of a conceptual theory or model here. What are we primarily interested in--utilization or outcomes? What is the main relationship they are hypothesizing for these two--that the poor should have more utilization? Of what kinds of services? They are missing a key reference which might help them frame this better: Cookson R, Doran T, Asaria M, Gupta I, Mujica FP. The inverse care law re-examined: a global perspective. Lancet. 2021 Feb 27;397(10276):828-838. doi: 10.1016/S0140-6736(21)00243-9. PMID: 33640069.

**- This paper presents an epidemiological analysis of income-based healthcare utilization (HCU) inequalities. To aid interpretation of group differences in HCU, we also include mortality as a comparative outcome. We agree with the editor the paper would benefit from a clarification of the study objective and main outcomes, why we now emphasize that group differences in HCU is our primary outcome measure, while mortality serves as a comparative outcome measure (see p.2 para.2; p.6 para.2; p.16 para.3; p.19 para.2; p.20 para.2). Our goal was not to test any hypotheses of how HCU affects mortality, but rather to answer the questions on how HCU inequalities develops over time. Despite lacking measures of need we believe we can draw valid conclusions on this. By assuming the existence of a (although not fully comprehended) relationship between need and mortality, and that this relationship remains relatively stable over the study period, we can infer trends in HCU inequalities: Since the highest income quantile utilized an increasing share of primary and outpatient care, while the odds of dying increased for the lowest income group, this indicates increasing disparities between need and HCU. This is now further clarified in the manuscript (p.19 para.2).**

**Thanks for pointing out this very relevant reference, which we now cite (p.5 para.1, p.19 para 3).**

ABSTRACT
Abstract Background:
Please expand details to provide wider context to the reader.
Line 15 -you state the study population included >15 year olds but in the main methods (line 81) you state 16 years – please clarify/revise for consistency.
Please move details of your study population to the methods and findings section of your abstract.
The final sentence should clearly state the study question.

**- This is now changed.**

Abstract methods and findings:
Line 19 – please include some of the sociodemographic variables to which you refer.
Please include any important variable factors that are adjusted for in your analyses.
Please clearly define the setting (national data?), number of participants whose data you include, and the main outcome measures.
Please quantify the main results with 95% CIs and p values. When reporting p values please report as p<0.001 and where higher as p=0.002, for example. Suggest reporting statistical information as follows ‘(OR 1.78, 95% CI [1.74,1.82[; p

**-Thank you for your suggested improvements. We have now implemented all of them in the text.**

AUTHOR SUMMARY
At this stage, we ask that you include a short, non-technical Author Summary of your research to make findings accessible to a wide audience that includes both scientists and non-scientists. The authors summary should consist of 2-3 succinct bullet points under each of the following headings:
• Why Was This Study Done? Authors should reflect on what was known about the topic before the research was published and why the research was needed.
• What Did the Researchers Do and Find? Authors should briefly describe the study design that was used and the study’s major findings. Do include the headline numbers from the study, such as the sample size and key findings.
• What Do These Findings Mean? Authors should reflect on the new knowledge generated by the research and the implications for practice, research, policy, or public health. Authors should also consider how the interpretation of the study’s findings may be affected by the study limitations. In the final bullet point of ‘What Do These Findings Mean?’, please describe the main limitations of the study in non-technical language.

The Author Summary should immediately follow the Abstract in your revised manuscript. This text is subject to editorial change and should be distinct from the scientific abstract. Please see our author guidelines for more information: <https://journals.plos.org/plosmedicine/s/revising-your-manuscript#loc-author-summary>

**- Author summary is now included.**

METHODS and RESULTS
Did your study have a prospective protocol or analysis plan? Please state this (either way) early in the Methods section.
a) If a prospective analysis plan (from your funding proposal, IRB or other ethics committee submission, study protocol, or other planning document written before analyzing the data) was used in designing the study, please include the relevant prospectively written document with your revised manuscript as a Supporting Information file to be published alongside your study, and cite it in the Methods section. A legend for this file should be included at the end of your manuscript.
b) If no such document exists, please make sure that the Methods section transparently describes when analyses were planned, and when/why any data-driven changes to analyses took place.
c) In either case, changes in the analysis-- including those made in response to peer review comments-- should be identified as such in the Methods section of the paper, with rationale.

**- No document exists, and this is now stated (p.10 para.1** )

For all observational studies, we ask the authors to indicate the following in the manuscript text:
(1) the specific hypotheses you intended to test,
(2) the analytical methods by which you planned to test them,
(3) the analyses you actually performed, and (4) when reported analyses differ from those that were planned, transparent explanations for differences that affect the reliability of the study's results. If a reported analysis was performed based on an interesting but unanticipated pattern in the data, please be clear that the analysis was data-driven.

**1) Investigated research questions are specified (p.6, para.2)**

**(2) the analytical methods employed are described in the method section, and the details of the specified models are in the S1 Appendix, and actual analysis code is available online at the link provided in the method section.**

**3) Post hoc sensitivity analyses are stated as such (p.9 para.3)**

As above (see under ABSTRACT), please quantify main results with 95% CIs and p values. When a p value is given, please specify the statistical test used to determine it.
When reporting p values please report as p<0.001 and where higher as p=0.002, for example. Suggest reporting statistical information as follows ‘(OR 1.78, 95% CI [1.74,1.82[; pLine 93 – please change ‘parenthesis’ to ‘parentheses’

**- These are now changed.**

Line 94 - Why are diabetes and CKD grouped together? Do these data refer to those with diabetic kidney disease or those with diabetes and separately kidney disease of any other cause? Or does it represent 2 groups of individuals some with diabetes and some with CKD? Please clarify/justify for the reader as it is currently unclear.

- **The disease group refers to the combined patient population of diabetes and CKD, in accordance with the disease categories used by the Global Burden of Disease study. We have now clarified this (p.7 para.2). Constituting ICD codes for each disease category are found in S1 Table.**

Line 152 – ‘(OR=1.04, 95% CI:1.04-1.05)’ please use commas to separate upper and lower CI bounds as hyphens can be confused with reporting of negative values (and to ensure consistency of statistical reporting throughout your manuscript).

**- Fixed.**

TABLES
Table 1 – presents very little data regarding the study population only total numbers and age, please include a comprehensive table detailing wider demographic details of the full cohort.

- **We agree this would enhance the table, and we have now added a more comprehensive demographic descriptions of the full cohort (e.g. percentage married, % Sweden born etc.)**

FIGURES
Please ensure that all figures are affiliated to an appropriate caption which clearly describes the figure content without the need to refer to the text.
Please consider avoiding the use of red and/or green to improve accessibility of your figures to those with colour blindness.
As above, when reporting 95% CIs please also report p values. Please report as p<0.001 or as p=0.002, for example.
Figure 1 – please indicate in the figure caption which factors are adjusted for. Where adjusted analyses are presented, please also include unadjusted analyses to help facilitate transparent data reporting. Please indicate the meaning of the black lines.
Could the chart be slightly more clearly/accurately labelled – DM & CKD, for exampleFigure 2 – what is the meaning of the shaded areas behind the red lines? What is the meaning of the dashed lines? What is the meaning of the horizontal thin red line? Please define for the reader. Please ensure consistent use of number formatting (for example, use of decimal places 5.0 Vs 5).

**- Thanks for the input on Figs above, we have now implemented all of it.**

Figure 3 – why does it differ in colour coding when compared to figure 1 despite detailing the same categories? Please revise for consistency (ideally avoiding the use of red and/or green).

**- To clarify for the reader that the outcome differs between Fig 1 and Fig 3 (ORs vs. annual change in log-transformed OR), we chose a different color.**
Figure 4 – In the figure title please revise to read ‘Healthcare utilisation (HCU)…’. What do the different (and sometimes overlapping) shaded areas represent? Please clearly define for the reader.

**- Fig 4 is now revised according to the proposals by reviewer #2, and the caption is updated.**

DISCUSSION

Please remove all sub-headings from the discussion such that it reads as a single piece of continuous prose. Please ensure that it is structured as follows: a short, clear summary of the article's findings; what the study adds to existing research and where and why the results may differ from previous research; strengths and limitations of the study; implications and next steps for research, clinical practice, and/or public policy; one-paragraph conclusion. Again, please avoid the use of sub-headings.
Line 213 – how is ‘severe’ defined and by whom?

**- We have now removed all subheadings, and stripped discussion from less relevant discussions on mortality, to fit the recommended structure. The text on “severity” of levels of care is now changed.**

SUPPORTING INFORMATION
TABLES
As above, please ensure that all tables (and figures) are affiliated to an appropriate caption that clearly describes its content without the need to refer to the text.
Please include p values where 95% CIs are reported (if not please clearly state the reasons why not).
Please indicate if your analyses are adjusted or unadjusted and where you present adjusted analyses, please clearly state the factors adjusted for. Please also include unadjusted analyses for comparison.

**-All suggestions above are now implemented. And unadjusted results, as well as results adjusted also for counties, are now included in the S4-5 Tables.**

S3 Table – please see reviewer #1 comments which we agree with. Please revise accordingly to improve clarity.
S5 Table – in the column headers please define the numerical within and outside of parentheses.

**-Both are fixed now.**

REFERENCES
For in-text reference callouts, please place citations in square parentheses.
Please see our website for other reference guidelines https://journals.plos.org/plosmedicine/s/submission-guidelines#loc-references
**- Fixed.**

Comments from the reviewers:

Reviewer #1: General comment

The paper focuses on income-based differences in healthcare utilization (HCU). Using a repeated cross-sectional register-based study design, authors analyze the relationship between income - comparing first and fifth quintile - and four dimensions (primary care, outpatient, inpatient, death) of healthcare use, controlling for main (five) diseases groups.

The topic of the paper and methodological approach is interesting; however, it presents a number of shortcomings in many ways.
The most serious drawbacks are the following.
First, authors include in their analysis both HCU and mortality, and this generate confusion about what is the topic that they are interesting to study. Analyzing the relationship between income and HCU or mortality need different approaches, theories, and scientific literature references. Furthermore, from reading the initial part of the paper it would seem the authors intend to study the relationship between HCU and mortality in a single model, on contrary they are treated as distinct outcomes. Looking at HCU in the five years prior to death (question 3: How do the low- and high-income groups differ in numbers of health care encounters ≤5 years prior to death?) does not justify the inclusion of mortality as a topic of the paper (questions 1 and 2: 1. How do low- and high-income groups differ with regard to HCU and mortality in 2017 and over time? 2. To what extent do income-related differences in HCU and mortality vary across disease groups?)...…

**- Thank you for highlighting this crucial issue. Indeed, the outcomes and their interrelationships should be clarified, and we have now attempted to do this by refereeing to HCU as main outcomes, and to mortality as comparative outcome (p.2 para.2; p.6 para.2; p.16 para.3; p.19 para.2; p.20 para.2). We do however believe that incorporating mortality as a comparative measure to healthcare utilization (HCU) significantly strengthen our study. By including mortality as a comparative outcome measure mortality, we can better interpret changes in income-related HCU differences. For instance, without mortality as a comparative outcome, it becomes more difficult to understand, for instance, the finding that the lowest quintile (Q1) has a 10% increased odds (vs. Q5) of neoplasm-related inpatient care but a 22% lower odds of outpatient care (2017). But knowing that Q1 has 54% increased odds of dying informs the reader that lower outpatient care is not due to lower need, but likely due to a relative underutilization. Furthermore, the fact that Q1's share of neoplasm related outpatient care decreases each year more than its share of inpatient care increases could misleadingly suggest to the reader that Q1 suffers less from neoplasms. However, when compared with the increasing ORs for mortality, we have good reasons to believe that the opposite is true.**

..At page 15, rows 282-283, authors' statement "…explaining the origin of the observed increase in mortality inequalities is challenging, and differences in HCU likely only play a partial role" it is probably true, but authors did not test this hypothesis in the paper. Moreover, pag. 13, rows 217: "…the large inequality in mortality rates were not reflected in correspondingly large group differences in HCU". To do it, they should have adopted a different analytical strategy: evaluate the impact of SES and HCU on mortality in a single model (for example using a SEM or GSEM models) to estimate direct and indirect effects.

**- Thanks for pointing this out. To make the discussion more readable and easy to follow we have now removed the discussions on HCU as a contributing factor to inequality in mortality. Although HCU is a likely related to premature death, any attempt to describe causality would, as the reviewer suggests, require us to include death and HCU in the same analysis. Mapping causal relationships in epidemiological observational studies is challenging and is beyond the scope of this paper. We would still like to thank the reviewer for the proposal to include income, HCU and mortality in for instance a Generalized Structural Equation Model. We believe this could be a useful strategy for future studies probing this relationship in more detail, why we now mention this in the paragraph on future directions in the discussion (p21, para.2).**

Second, as the authors themselves acknowledge, it is important to adjust for need when analyzing socioeconomic differences in HCU. The lack of this control variable in the models makes the estimates (differences between income quintiles) predictably biased. Treating the differences in mortality as a proxy for disparities in needs can not be considered an acceptable analytical strategy, since it uses an outcome, i.e. mortality, which is also the result of different needs related to socioeconomic inequalities to control for differences in health needs. This approach is tautological.

**- Yes adjusting for need is certainly important when analysing SES differences in HCU. All measures of need that we know have limitations, and unfortunately our national databases do not have survey data on needs to which HCU could be linked. Instead, we chose mortality as a comparative outcome. Like our approach, the relevant study recommended by the academic editor (**Cookson R, Doran T, Asaria M, Gupta I, Mujica FP. The inverse care law re-examined: a global perspective. Lancet. 2021) **inferred HCU inequalities by comparing HCU and life expectancy. We deem this to be a feasible strategy.**

**As we discussed (p.19 para.3), income differences in HCU are driven by several factors (e.g. differences in need, access, care seeking behaviour, etc), and here we are not able to determine the contribution of each. However, from the current results we can infer *trends* in HCU inequalities. Given there are any relationship between mortality and need, and given that this relationship is the same in 2004 and in 2017, the fact that mortality inequalities are increasing while Q1 utilize an decreasing share of primary and outpatient care, implies that HCU inequalities increased during the time period. This is now emphasized (p.19 para.2)**

Third, authors produce separate estimates for the main disease groups that account for the largest number of deaths in Sweden in 2017. This is certainly interesting, but they did not explain the differences observed in the results between the diseases groups, reporting only the estimated values. So, what is the utility of doing these analyses?

**- The utility of doing disease specific analysis is that it allows us to identify which disease (at which level of care) suffered from largest HCU differences, and thus tells the reader where the largest potential for improving health care equity could be found. So, based on scale (i.e., high prevalence or disease burden) and tractability (large HCU inequity), we would deem e.g., increased utilisation of primary and outpatient care for neoplasm by low income groups to be a particularly promising target, which we now emphasize in the discussion (p.17 para.3 ; p.21 .para 3). Furthermore, by identifying similar patterns of inequality gradients in HCU and mortality among independent patient populations strengthen our conclusion. However, we agree that the variation in the inequality pattern across disease groups indeed is interesting and could be investigated further, why we have added discussion on this (p.17 para.3)**

Fourth, the Discussion paragraph is very fragmented, making it difficult to understand if and to what extent the hypotheses are corroborated and how to interpret the results in the light of the aims of the paper. For example, if the theoretical background of the paper is the fundamental cause theory, it should be introduced in the first part of the paper and the results should be discussed in the light of this theory.

**- We agree that the discussion should be clarified by discussing the results in the context of the study aim. We have now deleted parts of the discussion that are more distal to the research questions (e.g., the section on trends in mortality, and section of HCU as an explanation of increased mortality inequalities), and instead elaborated on the results in the context of the disproportionate care law, the trends in HCU inequalities, and the potential causes for HCU inequalities (p.17 para.3; p.19, para. 3).**

Other shortcomings

Pag. 5. The study population comprised all individuals 16 years and older, but, as we know, most young people enjoy good health, as we can also see from the average age in the use of different types of health services (table S3), therefore the analyzes could be conducted on a population with a different age range.

**- Given our aim to describe inequality trends in as large portion of the Swedish population as possible, we chose to include all ages for which data were available. HCU and mortality occurs of course among young people too. Importantly, since we group the study population by income as stratified by birth year, no systematic biases are introduced by including younger ages.**

Pag. 6. Authors should discuss the reliability of the income data used dataset, as we know this kind of data is difficult to collect with accuracy.

**- Income data relies on taxation records, and are deemed to be reliable. Statistics Sweden write that *(“The statistics are compiled using the Income and Taxation Register (IoT), which is based on administrative register data from the Swedish Tax Agency (taxation and income data), the Swedish Social Insurance Agency, the Swedish Board for Study Support, the Swedish Pensions Agency, the Swedish Agency for Government Employers, the Swedish Armed Forces, the Swedish National Board of Health and Welfare, and the Swedish National Agency for Education. Since these data are primarily used for other purposes, there is a high probability that errors are detected and corrected in the source registers. Therefore, the statistics are generally considered highly reliable and have good coverage regarding various types of income*”. (**[**https://www.scb.se/contentassets/894d4afa900f4123aece341cd10e59e2/he0110_kd2_2019_jl_210127.pdf**](https://www.scb.se/contentassets/894d4afa900f4123aece341cd10e59e2/he0110_kd2_2019_jl_210127.pdf)**) . As mentioned in the Discussion, null-taxers are removed, since they constitute a heterogeneous group not easily classified in a SES context. We have also added a more extensive description of the income data to the appendix (S1 Appendix).**

Pag.13, rows 212-213. Authors state: "For all diseases combined, the 213 more "severe" the outcome, the larger the inequalities". How is the degree of severity of the health service used established?

**- We agree this is an unclear expression and has changed the sentence.**

Pagg. 16-17. Authors did not clearly explain because in a universal healthcare they observe differences in HCU on the basis of income levels. For example, they wrote: "Although financial barriers to access to care have been largely removed in modern welfare states, the so-called 300 inverse care law suggests that "availability of good medical care tends to vary inversely with the need of the population served" ([1] p 459, Hart 1971). An increasing number of private health providers and the free right to establish care centres in any location has in recent years reduced the number of child and maternal care units in poorer areas where the need is greater (24). In addition, availability is further skewed in favour of high SES HCU due to the increase in private insurances and out of pocket health care spending". First, why should people take out private insurance in a universal healthcare system like the Swedish one? This point should be further explored. Second, authors did not control for these possible confounding variables in their analysis. Third, examples about the inverse care low refer to healthcare services which are not related to groups of diseases considered in the paper.

**- Typically individuals have private insurances through their employment, and a main motive is to bypass the waiting lists in publicly funded care. The percentage of adult working population with private insurance was 13% in 2017 (**[**https://www.commonwealthfund.org/international-health-policy-center/countries/sweden**](https://www.commonwealthfund.org/international-health-policy-center/countries/sweden)**). We think that further explorations of the motives for private insurances would be too remote from the main objective of this paper. Thus we think it suffice to mention the existence of private insurance as a factor that most likely further increase the observed phenomena of smaller group differences in HCU and larger differences in mortality. Regarding the second point, as stated (p.19 para.3) we don’t have microdata on private health care utilization, and thus we cannot control for these. For the third point, although maternal and (some) of the child health care is contributing to the all-disease group (why we keep this reference), it is true that other types of care would more directly impact HCU trends we investigate here. Hence, we have now added several references e.g. on how virtualization of primary care is unproportionally used by high SES groups, etc (p.19 para.3).**

Pag. 22. S3 Table caption is partial, and content is not clear. For example, what does Full population, N in the fourth column mean? The content is not an absolute value but a rate, therefore N is wrong here.

**- Thanks for pointing this out. The table caption is now clarified.**

Reviewer #2: The manuscript describes income differences in health care utilisation (HCU) and mortality in Sweden between 2004-2017, and the development in these over time. It finds small differences by income in primary and outpatient care, but large and rising inequalities in inpatient care and mortality. They also display interesting differences in the social gradient in HCU and mortality by disease group for five major groups. All in all, I found the manuscript an interesting read which should be relevant for a broad audience. Here are a few comments.

1. Minor points on the study population: The population specification in line 81 should be residents in 2004-2017, a typo. Table 1 should have a title that describes its content, i.e. size and age of study population.

**- Thanks, this is now clarified.**

2. They utilise primary care data that they have collected themselves from counties, as there is no central register. This is a major endeavour and contributes to the novelty of the study, which could perhaps be highlighted in the introduction; i.e., due to unique data, this is the first study to consider income differences in primary care use in Sweden and compare it to other HCU. Given this novelty, the highly interested reader would also want to know more about this dataset, perhaps in the supplementary material, including whether you believe it is reliable/complete and whether it is the basis for disbursements. Especially, I am curious which counties contributed how many years, and which did not. The authors have anonymised this information, which is a shame for both replication purposes and interpretation, because Swedish counties are very different wrt. degree of centrality, immigration, health care availability and inequality. If anonymisation is a requirement, that should be stated. They should include some summary statistics showing whether the included county-years differ from the total population (on these dimensions, and also in levels and differences in specialist HCU and mortality). They should display the main results (e.g., Fig. 4) with the same county-years as their primary care sample for the other outcomes, possibly as supplementary material. Furthermore, they should return to this in the discussion. A strength is that they look beyond primary care. A weakness is that primary care data coverage is incomplete, they should speculate on how that may have affected the results, and optionally call for a national register which could improve coverage and data quality for future research. Swedish policy-makers need to hear this from the researchers, it is one of the few aspects where Swedish data is not to the standard of other Nordic countries.

**- Thanks for pointing out the uniqueness of the data base we have compiled. We agree, and have now highlighted the novelty of using primary care data in the introduction (p. 6 para. 2).**

**In the manuscript we acknowledge the limitations of the primary care data, such as the unknown and most likely changing coverage rates. We also discuss the potential implications of deficient primary care data on the results (p. 20 para. 4). In S2 Table, we now also mention that the data does not necessarily constitute the basis for disbursements.**

**Included counties are not anonymized but are referred to by their identification digits as used by Statistics Sweden and NBHW. For clarity we have now also added the actual names S2 Table).**

**The reviewer ask for a summary table describing the county years with regard to e.g. centrality, health care availability etc. We have now added information on the cohorts, e.g., a measure of inequality (the income ratio Q5/Q1, Table 1). Regarding measures of centrality and health care availability, we unfortunately don’t have access to such data. However, given that, as explained in the methods section, we do not have complete or known coverage rates for each county year, a detailed sociodemographic description would not substantially improve the readers understanding of the results.**

**Given the very extensive description of HCU trends, redoing the analyses by including only the county year for which primary care data is available, and stratify the new population in new income groups etc, would double the number of analyses, and would require substantial work that to in our opinion unlikely would shed new light on the research questions, why we are reluctant to conduct such. However, instead we have ran a sensitivity analysis where we in addition to previous control variables also adjusted for county, in an attempt to control for county effects caused e.g. by varying coverage rates. This mainly reduced income-related differences in primary care (see the added rightmost panels of S4-5 Tables) and is now mentioned in the discussion (p. 19 para. 4)**

**To sum, at this point, given the available data, we believe the best way to handle the deficient primary care data is to 1) incorporate socioeconomic variables as control factors in the regression analysis, 2) be careful when interpreting the absolute rates of primary care utilization, and 3) triangulate the primary care results by incorporating complementary data on specialized care utilization, 4) discuss potential implications of varying coverage rates of primary care data, and 5) conduct post-hoc sensitive analyses adjusting for counties.**

**Finally, we fully agree on the usefulness of such a national register of primary care data akin to what is available in e.g. Norway and Finland, and we have now called for this in the discussion (p.21 para. 1)**

3. Relatedly, in their data statement they state that «Data are available from the Swedish National Board of Health and Welfare and Statistics Sweden». Does this also cover the collected primary care data? What are the prospects of also making that data available for other researchers? Could you give a reference number for the data orders to the National Board and Statistics Sweden, so that other researchers with the necessary permits could order the same?

**- Swedish National Board of Health and Welfare harbour the raw primary care data and maintain the key for pseudonymized individual identifiers enabling linkage to other register data. To use the primary care microdata for research projects other than what current ethical permission regulates would require approval from the regional databases. There has been discussion of including primary care data in a national health register by NBHW, but until this is in place, each research project are requested to apply for data directly from original data owners, and have them send their data to NBHW where it is compiled and anonymized before sending it to us.**

4. I find the methods section under-explained and at times confusing, and suggest a larger methods section, perhaps in the supplementary materials. Ideally, the methods should be well enough described for other researchers to be able to replicate the study and assess the comparability with other studies. I encountered an empty repository when following the provided link. Seeing the code could have helped, but ideally the manuscript should be self-explanatory without needing to read code. They have used what they refer to as family income. It is not clear what constitutes a family and why household income was not used, which limits comparability to e.g. Chetty et al. (2016) and Kinge et al. (2019). The equivalence scale used should be specified. Equations used for the regression analyses should be stated. In the logistic regression, the method for computing standard errors is not specified, clustering on individuals would be a reasonable choice since the same person is observed multiple years. It is unclear how they regressed log transformed ORs on time, whether they used individual year dummies or parametric time trends.

**- We apologise for not yet have provided the code. It is now uploaded.**

**A detailed description of family income is found in the SCB (Statistics Sweden) variable lists. In our experience** **the choice of income variable have only a minimal impact on the results. Initially, in the original preparation of the manuscript, we used DispInkK. Now we have changed to DispInkKE04 (and DispInkKE prior to 2004), with only very minor effects on the results. We changed to DispInkKE04 since SCB refers to it as “..a recommended method often used in the research world and also in the statistics produced within the EU and other national statistical agencies”. SCB further explain the equivalence scale: “*… the disposable income per consumption unit is obtained by dividing the sum of all disposable income of the members included in the family by the family's total consumption weight. Negative values can occur. Example: A family in 1993, consisting of two adults, one child 0-3 years, and one child 4-10 years. The family's total consumption weight = 1.92 (two adults) + 0.56 (child 0-3 years) + 0.66 (child 4-10 years) = 3.14. The family's disposable income = 300,000 kr. The individual's (adult) disposable income = 300,000 / 3.14 = 95,541 kr.”***

**We have referenced the full description of the variable in our S1 Appendix, where the equivalence scale is described.**

**Equations used for the regressions are now included (S1 Appendix).**

**We have now clarified that we used parametric time trends (p9. para.1)**

5. I would read this paper with an interest in income differences in the entire Swedish population. However, the paper quickly develops into a comparison of two outlying groups, Q1 vs. Q5. Instead of Fig 1, I would want to see the ORs for all groups Q1-Q4 compared to Q5. In Fig 2, I would want to see the development in all five quintiles. It is a very different inequality if all groups Q2-Q5 have similar levels and Q1 is an outlier than if it is Q5 that differs from the rest. It is also important for our understanding how each group develops across time.

**- The paper aims to compare the lowest with the highest income groups, clearly stated in abstract and introduction. This is now further clarified in the aim (p.2 para. 2; p.6 para 2)**. **When depicting trends (Fig 2 and 4, table S3) we also included the total Swedish (adult) population. The set of analyses are extensive, and a large set of data points are conveyed in a manner that aims to best answer our research questions. However, we understand the need of putting in the lowest and highest income groups in perspective, and therefore provide a supplemental figure displaying HCU and mortality rates for each income quantiles over time (S2 Fig).**

6. I would encourage the authors to rely on their graphic depictions of each quintile's development across time, rather than running the log-transformed time series analysis. The additional analysis seems unnecessarily complicated, and again narrows the focus to Q1 vs Q5 developments. I also struggle to trust and interpret the estimates. Take their estimate for all diseases, they find a yearly change in log ORs of -0.006 for outpatient care, and 0.012 for inpatient care (see table S4). This is a complicated size, they do not provide any direct interpretation or unit of measurement. It could seem like the negative change in outpatient care (i.e. decreasing inequality) is half the positive change in inpatient care (i.e. increasing inequality). However, when eyeballing Fig. 2, I see a clear increase in inequality in inpatient care and a bumpy but flat development in inequalities in outpatient care. In fact, in table S4 we can also read that the Q1/Q5 OR slightly increased from 1.038 to 1.042 in 2004-2017 in outpatient care, although the time trend estimate is a precise and sizeable negative.

**- Again, study aims to characterize** **developments of difference in HCU for Q1 vs Q5, and thus we are reluctant to substitute Fig 3 by each quintile’s development.**

**The rationale for conducting regression analysis on the log-transformed time series is twofold. Firstly, utilizing the entire time series (i.e. 14 data points) instead of solely relying on the first and last data points provides more robust estimates. Secondly, presenting the beta parameters in their log odds scale, rather than in an exponentiated or "OR" scale, ensures that the magnitude of the regression parameters are comparable across all variables. Hence, as the reviewer points out, the negative change observed in outpatient care (indicating decreasing inequality) is exactly half of the positive change observed in inpatient care (reflecting increasing inequality). However, if the results were exponentiated, this intuitive linearity would be lost. Instead, if we would have exponated the regression parameters we would have a rather complex unit of the outcome: OR (T1 vs T1+1) of OR (Q1 vs Q5).**

**Instead we provide both ORs at the endpoints (2004 and 2017), as well as the regression parameters of yearly change, along with graphical representations. We believe that this is the best way to help the reader to understand the time trends.**

7. Figure 4 is difficult to appreciate. It is not clear why the authors are now also interested in Q3, I would again like to see all quintiles. They have changed the reporting to percentage change, however I suspect the estimates are still based on ORs. They lack a justification for why Fig 4 is displayed differently than Fig 2, having the same presentation on this outcome would make the reading easier. This should be the absolute development of all quintiles, and if they wish, add the Q5 vs Q1 OR comparison. Having all the scales going in +/- 50% makes the figures less readable. In general, their conclusion is that they don't find significant income differences in primary or inpatient care in any disease group 5 years before death, but sizeable under-utilisation of outpatient care for most disease groups. Also displaying overall HCU 5 years prior would make this point more clearly, and improve power (which seems to be a problem in this analysis). This finding should be picked up more in the discussion. What characterises outpatient care in Sweden, why is it so under-used by low-income groups with short remaining life expectancies, and could improving outpatient HCU reduce mortality for low-income groups?

**Thanks for pointing this out, and we agree that the analysis of number of encounters <5 yrs prior to death should be described in more detail.** **In the process of revising the manuscript, we recognized that given the nature of the outcome (# encounters as count data rather than continuous data), it is more appropriate to run negative binomial regression instead of our initial ordinary least squares (OLS) regression. We have now rectified this. The new outcome is Rate Ratios, and the analytical procedures are now clarified in the Methods (p.9 para.2 ) and in S1 appendix.**

**We agree with the reviewer that presenting Fig 4 in the same way as Fig 2 would make the reading easier, why we now have changed to this.**

**Again, we want to stick to the goal of the paper to compare Q1 and Q5. All income groups are certainly interesting, but the number of results to present and discuss is already now very extensive, and we believe adding three times as many group comparisons would simply be daunting.**

**We chose to display Fig 4 with shared right y-axis to facilitate visual comparisons of income-related differences across levels of cares and disease categories. Reader is referred to S5 Table for exact numbers.**

**The point of displaying overall HCU is well taken, and is now implemented (Fig 4).**

**We have also added text in the discussion on putative explanations of the results Fig 4 (p.19 para 3-4).**

8. In the discussion, the causes and consequences of differences in utilisation by disease group seems underdeveloped. To me, this is one of the most striking findings in the paper. Low-income groups seem to under-utilise primary and outpatient care for neoplasms and chronic respiratory diseases, but use them frequently (and increasingly so) for neurological diseases and diabetes. They see the doctor, but perhaps not for the right reason. A pattern emerges where these groups use health care too little for prevention, resulting in an over-usage for treatment. Could GPs use the consultations with these groups that are requested for diabetes and neurological disorders to prevent and detect cancer and COPD risks, and through that possibly affect later need for inpatient care and mortality risk? This could be one point for future studies.

**- Given the comprehensive data set and numerous outcomes analysed, striking a balance between a clear take-home message and including interesting findings has been challenging. Although we believe that a thorough discussion on the variations in income-related HCU differences across disease groups is beyond the scope of this paper, we agree with the reviewer that it deserves to be discussed in some more detail. We are grateful for the reviewer’s observation that low income groups seem to suboptimally use primary and outpatient care, and have now included this in the discussion. (p.17 para.3)**

9. In the discussion on causes of increasing inequality in mortality, the authors write that inequalities tend to increase with declining overall mortality levels (lines 272-273). The authors should add an explanation of why it is the case that inequality increases as mortality declines, and perhaps discuss whether this holds in all stages of the epidemiologic transition, including the fourth stage of decline in degenerative diseases (Ohlsansky & Ault, 1986). The main point in their reference (Mackenbach, 2019) is that SES differences tend to remain (not increase) even as overall mortality declines, but also that there are large variations in this relationship between countries and across time periods. Increased socio-economic selection is one factor he mentions that may cause this, as social mobility and increasing returns to education makes income more predictive of general skills and abilities. Another is that taking advantage of improvements in public health requires ability for behavioural adaptation and health literacy. Some explanation/moderation is needed so that the co-development does not appear to be deterministic.

**- To improve clarity of the discussion and keep it aligned with the main aim of the paper, we have trimmed the discussion on mortality inequalities and their causes, and the passage in the original draft mentioning declining mortality rates as an “algebraic” driver of increasing inequalities everything else equal, is now removed. (As a side note, originally, we just pointed out that for a given absolute rate difference, say 1 per 1000 individuals, in a scenario of high general rates, say Q1 15 per 1000, and Q5 14 per 1000, this yields a relative difference of 7%. Compared to a scenario with lower mortality rates, say 11 per 1000 for Q1, and 10 per 1000 for Q5, the relative difference is 10%. Thus, despite constant absolute differences, relative differences tends to increase when overall rates decrease.) But again, this reasoning is now omitted for the sake of clarity.**

10. The title of Table S3 suggests that it presents rates, but it also presents average ages. The ages can be dropped or need to be stated in the title. In the column headings, they use N for rates which is unfortunate; write out rate instead. N can easily be confused with number of observations, and they use N for this in Table 1.

**- Thanks for pointing this out. The caption of S3 is now clarified**.

11. I would be interested in all ORs Q1-Q4 in Table S4 and S5, not only Q1 vs. Q5 and Q1 and Q3 vs. Q5 respectively.

**- We strive to capture the most relevant information as concisely as possible, and already struggle with very large tables. If we would include another 3 income groups, S4-5 Tables would expand manyfold. It would also be challenging to discuss and refer to all additional results pertaining to all income groups. However, we hope and believe that our new S1 Fig will sufficiently address the need to overview trajectories of rates among all income groups.**

References:
Chetty, R., Stepner, M., Abraham, S., Lin, S., Scuderi, B., Turner, N., ... & Cutler, D. (2016). The association between income and life expectancy in the United States, 2001-2014. Jama, 315(16), 1750-1766.
Kinge, J. M., Modalsli, J. H., Øverland, S., Gjessing, H. K., Tollånes, M. C., Knudsen, A. K., ... & Vollset, S. E. (2019). Association of household income with life expectancy and cause-specific mortality in Norway, 2005-2015. Jama, 321(19), 1916-1925.
Mackenbach, J. P. (2019). Health inequalities: Persistence and change in modern welfare states. Oxford University Press, USA.
Olshansky, S. J., & Ault, A. B. (1986). The fourth stage of the epidemiologic transition: the age of delayed degenerative diseases. The Milbank Quarterly, 355-391.


Reviewer #3: By linking multiple datasets (Longitudinal Integrated Database for Health Insurance and Labour market Studies, National Patient Register, Cause of Death Register), the authors tried to assess time trends of income-related differences in use of different levels of health care in relation to mortality between the years 2004 and 2017. The three research questions of the study are 1)
How do low- and high-income groups differ with regard to HCU and mortality in 2017 and over time? 2) To what extent do income-related differences in HCU and mortality vary across disease groups? 3) How do the low- and high-income groups differ in numbers of health care encounters ≤5 years prior to death? Overall, I think the study topic is of importance and the results are relevant for policy making. However, I think the study methods would require some improvements. Below are my specific comments.


1. The data are not fully publicly available, which may not comply with PLoSMed's policy.

**- This is unfortunately unavoidable when reporting on integrity revealing microdata from population registers. However, the original databases is referenced, and their contact information is now added (S2 Table).**

2. Line 47, and 53-55, instead of quoting the original sentences, I think it would be better to paraphrase the original sentences or summarize the main idea of the original sentences, especially when the quoted sentences are very long.

**- We agree and have now paraphrased and condensed the citations.**

3. Line 43-78: The introduction could benefit from providing more context on the global issue of SES disparities and their effects on health care access and outcomes. This would allow readers to better understand the broader implications of the study.

**- Thanks for this suggestion. In the introduction we have now added an expanded outlook on SES disparities in HCU, including a new reference (p.5 para.1).**

4. Line 81, between 2004 and 2017?

**- Corrected**.

5. Line 108-110, are the income metrics listed here comprehensive and representative of people's wealth? I am thinking real estate properties people have or other capital assets and may not yield capital return.

**-No, income solely refers to income and not wealth. This is because in Sweden, taxation is based on income rather than wealth. Unfortunately, official national statistics on individual wealth in Sweden are not available beyond 2008.**

6. Line 113. Also, I think income level is only one of the many contributing factors to SES level. Income level cannot even represent people's wealth accurately. Social status (such as job type) and education level are also important contributing factors to SES level. I think it's a bit inaccurate to use income alone to define SES. I would suggest the authors just use income level instead of SES level.

**- We fully agree that SES ultimately is not reducible to income, although the most common indictors of SES (income, educational level, occupation) typically are highly correlated. The advantage of using income rank when studying inequality trends is that it allows for interpretable, equally sized SES groups over time. However, we agree income levels is a more precise description, and have now changed the wording to that.**

7. Line 126-127, I think the model also needs to adjust for people's health status or perceived health status because that can be an important bias covariate of the relationship between income level and HCU or death.

**- We fully agree with the reviewer about the importance of having an independent measure of individuals' health status to accurately analyze HCU inequalities, as we extensively discussed in our Discussion section (p.18 ,para.2). However, we are not aware of nationwide microdata that could be used. Death is therefore here employed as a comparative outcome measure. We agree this has been unclear in our paper, and have attempted to clarify this in the new version (p.2 para.2; p.6 para.2; p.16 para.3; p.19 para.2; p.20 para.2)**

8. Figure 2: The y-axis on the left should be percent or rate? Percent and rate are quite different concepts.

**- It is percentages (number of cases per 100 individuals, or rates per 100). When we tried using rates per 100 000, this yielded larger numbers rendering more white spaces between the panels, which decreased the visibility of the graphs. We have now clarified this in the figure caption)**

Any attachments provided with reviews can be seen via the following link:
********

__________________________________________________
In compliance with data protection regulations, you may request that we remove your personal registration details at any time. (Use the following URL: https://www.editorialmanager.com/pmedicine/login.asp?a=r). Please contact the publication office if you have any questions.
