# Supplementary material for: Income-based differences in healthcare utilization in relation to mortality in the Swedish population between 2004–2017: A nationwide register study
Source: PLoS Med. 2023 Nov 16;20(11):e1004230. doi: 10.1371/journal.pmed.1004230 (PMC10653442; doi:10.1371/journal.pmed.1004230)
Supplement: Attachment — Submitted filename: ReplyToReviewers2.docx [file pmed.1004230.s011.docx]

Dear Editor and reviewers,

Thanks for reviewing our manuscript once again. We have conducted the new sensitivity analysis and carefully considered all your comments, and we provide a point-by-point response in (**bold**) to each of them below:

---------------------

Requests from the editors:

GENERAL
Thank you for your detailed responses to previous editor and reviewer comments. Please see below for further comments which we require you address in full.
Throughout, where possible including in the figure and table captions please refrain from using the word disease (exception would be the ICD-10 coding) and replace with an alternative, perhaps co-morbidity or similar.

**-We authors have discussed your suggestions, but we are not sure we understand why and how to refrain from using the word disease. As described in the methods section, here we use the same disease categories as those used in the Global Burden of Disease study (https://vizhub.healthdata.org/gbd-compare/), where these are commonly referred to as 'diseases'. After reviewing their website (**[**http://ihmeuw.org/66vt**](http://ihmeuw.org/66vt)**), we noticed they use the plural form for diseases, and ‘neurological disorders’ for neuro. We have now revised our terminology to match theirs.**

**We report on number of individuals registered with any ICD-10 code pertaining to each of the broad disease groups. Thus, individuals could very well have only one of the many ICD codes, rather than ‘co-morbidities’. This should be clear from reading the method section (p. 9, para. 1). Please let us know if we misunderstand your suggestions, and if you have alternative word other than co-morbidity.**

*** Reviewer #2 has asked for the inclusion of additional analyses (please see below) which have the potential to alter the interpretation of your data and for that reason we have requested a further major revision ***

**-** T**he additional analysis has now been conducted, with results that are essentially identical to the original ones (see the new S2 Fig in comparison to Fig 2). Please see our reply to reviewer #2 for further details on this point.**

DATA AVAILABILITY STATEMENT
Thank you for including contact details, as per my previous request, in the main manuscript for Swedish National Board of Health and Welfare and Statistics Sweden. Please only include these in the manuscript submission form and remove from the manuscript itself.

-**Thanks for clarifying, this is now done.**

Please also place the details you provide in table S1 in the relevant part of the manuscript submission form. Please indicate that these are for access to primary care data, as we understand things.

**-Thanks, we will provide this in the manuscript submission form.**

In the event of publication these details will be compiled as metadata.
In the manuscript submission form, please replace ‘Wellfare’ with ‘Welfare’.

**-Fixed.**

COMPETING INTERESTS
All authors must declare their relevant competing interests per the PLOS policy, which can be seen here:
<https://journals.plos.org/plosmedicine/s/competing-interests>
For authors with ties to industry, please indicate whether any of the interests has a financial stake in the results of the current study.

ABSTRACT
In the last sentence of the Abstract Methods and Findings section, please describe the main limitation(s) of the study's methodology.

**-We have now added a sentence describing the main limitation in the abstract.**

AUTHOR SUMMARY
Thank you for including an author which reads very nicely.
Line 56 – suggest ‘data’ instead of ‘microdata’

- **Thank you. The wording is now changed.**

Line 59 – not sure ‘diminishing’ is the right word here, it makes the sentence a little unclear. Do you mean that despite having the highest mortality rates, those in the lowest income quintile access healthcare the least? Please revise for clarity.

**- Yes, that’s what we mean, and that this is reinforced over time. ‘Diminishing’ is now replaced by ‘decreasing’.**

Line 63 – what do these findings mean? - please describe the main limitations of the study in non-technical language. ‘lack of adjustment for individual healthcare need’ at line 64 could form part of this point.

- **This is now added.**

METHODS
Line 189 – please remove the funding statement and include only in the manuscript form when you resubmit the manuscript. In the event of publication it will be compiled as metadata.

**- Fixed.**

Lines 191 -193 (STROBE statement) please move to the beginning of the methods section.

**- Fixed.**

TABLES
Table 1 – characteristics of the study population still lacks adequate sociodemographic details which should be available when considering the data bases used. It is also not very accessible and presents selected data and not complete data. Please revise.
Please review our currently published articles on our website <https://journals.plos.org/plosmedicine/> for suitable examples.

**-In Table 1, we describe the cohorts in terms of the sociodemographic variables utilized in our analyses (e.g., birth country, age, and marital status). Additionally, we show the income ratios since this is relevant for the discussion on limitations. Given our stratification of income groups by birth year and sex (as detailed in the methods on p.8, para 2), we think that including sex-distributions of the groups would be redundant. Hence we have tried hard to think which relevant sociodemographic details that one should include (education, income, sex etc), but we could not conclude on any. Could you please specify which, if any, that you lack?**

**Regarding readability, Table 1 is designed to align with the main results table (S3 Table) to facilitate easy comparison. As a result, we present the income groups in columns and the years in rows. We've chosen to display only four distinct year categories: start-, mid-, and end points, and an aggregated ‘all years’. This was done to maintain clarity and avoid overwhelming the reader with another (14-3)*2 = 22 rows of near identical info.**

**We have looked at several PLOS medicine articles and once again revisited the PLOS medicine guidelines for table creation. Based on this, we've made our best efforts to enhance the clarity of Table 1 by providing more detailed explanations in the legend, merging cells, etc. Again, we attempted to strike the balance between readability and completeness. If further modifications are required, could you please give more specific guidance on what you would like to change?**

FIGURES
Please avoid the use of the color red and/or green to make your figures accessible to those with color blindness.

**-In the last revision we were asked to consider not using red-green combination. We followed this advice and changed the colors to red-blue instead, as this combination typically is considered ’color blind friendly’ (see** [**https://www.tableau.com/en-gb/blog/examining-data-viz-rules-dont-use-red-green-together#:~:text=For%20example%2C%20blue%2Forange%20is,palette%20designed%20by%20Maureen%20Stone**](https://www.tableau.com/en-gb/blog/examining-data-viz-rules-dont-use-red-green-together#:~:text=For%20example%2C%20blue%2Forange%20is,palette%20designed%20by%20Maureen%20Stone)**). We also use different thickness of the blue lines and the red lines. A quick look at the PLOS Medicine website on articles using multi-color figures also confirms that red or red in combination with blue are used previously (see e.g., for recent examples:** [**https://journals.plos.org/plosmedicine/article?id=10.1371/journal.pmed.1004261**](https://journals.plos.org/plosmedicine/article?id=10.1371/journal.pmed.1004261)

[**https://journals.plos.org/plosmedicine/article?id=10.1371/journal.pmed.1004273#**](https://journals.plos.org/plosmedicine/article?id=10.1371/journal.pmed.1004273)**)**

**Regarding Fig 1, the four bars have incremental decrease in luminosity, corresponding 1:1 to the luminosities of Fig 3. In other words, a greyscale print of Fig 1 would render identical range of grey bars for Fig 1 and Fig 3. While we referred to the PLOS Medicine guidelines for figure preparation, we did not find specific details about required or recommended color schemes, so please let us know if there are and we will make the necessary adjustments.**

Figure 1 – please define OR in the caption ‘Adjusted odds ratio (OR)…’
Figure 2 - please define OR in the caption ‘Adjusted odds ratio (OR)…’
Figure 3 - please define OR in the caption ‘Adjusted odds ratio (OR)…’ and as per my previous request please define all abbreviations used to categorise co-morbidities.

**-We have now defined OR in the captions (Fig 1-3) and added explanations of the abbreviations of the disease categories also in Fig 2.**

DISCUSSION
*** The language and grammar of the discussion needs careful attention as many sentences are unclear and appear partially written. Please check carefully throughout and amend as necessary. Specific points are detailed below but the list is no exhaustive ***

Line 312 – please remove ‘treatment in’
Line 313 – suggest ‘Further research exploring the benefit of allocating resources towards prevention and early treatment…would be beneficial.’ Or similar.
Line 315 – ‘rather than on less treatable neurological disorders.’ This could be misinterpreted and cause offense as it suggests neurological disorders aren’t worth investment. Please revise/remove.
Line 355 – ‘Despite we did not adjust HCU for need…’ please revise as this is grammatically incorrect, perhaps instead, ‘Despite not adjusting for HCU need…’ as we understand things.
Line 356 – ‘Assumed the existence of any…’ it is unclear what you are trying to say here, please revise for clarity Line 353 – ‘To better determine this, future studies should use proper control for group differences in medical need.’ Please use an alternative word to ‘proper’ which is unscientific and vague.
Line 426 – as above, please remove the data availability statement from the main manuscript and include only in the manuscript submission form. In the event of publication it will be compiled as metadata.

**-Thank you for all suggestions. We have now rectified all the remarked texts. We have also reviewed the language in the Discussion.**

REFERENCES
For web references please replace the term ‘cited’ with ‘Accessed’

**- Fixed.**

SUPPORTING INFORMATION
S4 and S4 Tables – thank you for including these tables which are very clear. Please also define ‘confidence interval (CI)’ in the footnotes of both.

**- Thank you. The footnotes are now changed.**

S1 Fig – as above please refrain form using red and/or green to improve accessibility of your figures to those with color blindness.

**-The color maps selected for S1 Fig were chosen to be color-blind friendly by avoiding the problematic red-green combination. Please see our previous response regarding the colors, and let us know if you disagree with our motivation for keeping current colors.**


Comments from the reviewers:

Reviewer #2: Major comment:
The authors have responded adequately to my comment 1 and to some of the issues raised in my comment 2. However, I wanted to see the results on levels and trends in HCU and mortality for the same sample of counties where they have primary care data throughout the period. Simply put, the trends you see in primary care usage may be due to sample changes rather than a development in health care usage, unlike the specialist care case where statistics from the whole country are available throughout. The difference in levels between primary and specialist care seem to be partially due to the county composition, as controlling for county has an impact. They commented on that this matters when considering encounters during the last 5 years prior to death, but also in the main analysis, the 2004 estimate for primary care changes from 1.11 when not adjusting for county to 1.30 when adjusting, so that the Q1/Q5 ratio is actually higher than for inpatient care that year (where it is 1.22). These results from model 3 in S4 need to be discussed. It is of primary interest for the research question whether the difference they see between the income gradients in primary care and inpatient care use is in fact an artifact of sampling differences.

-**We have now conducted this additional sensitivity analysis, using the same subsample used for analysis of primary care data to analyze specialized (outpatient and inpatient) care and mortality. The analysis is mentioned in the Methods (p.9, para. 2), and the results are presented in the supplements (S2 Fig), and in the Results (p. 11, para. 3). As evident when comparing to Fig 2, the time series of ORs of specialized care and mortality are almost indistinguishable from the result in the original full national sample. Thus, the reported differences in ORs for primary care and for specialized care or mortality are not due to differences in the sample populations.**

**We now refer to the results of model 3 in the Results (p 13., para. 3) and discuss the OR findings in the Discussion (p. 17, para. 1), where it nicely corroborates the findings of RRs being discussed.**

Minor comments:
I commend the authors for thorough responses to my comment 3 and 4, and especially the updated method section and supplement S1 gave a much clearer picture of what they did. Now that I understand that they have repeatedly simulated linear trends, a question that I wish they could address in the paper is whether the developments are well proxied by linear trends. Stating that they have looked at the plots year-by-year and visually inspected that it looks linear could suffice.

**- We agree that our choice of using linear model should be motivated, why we now added this to the methods section (p. 9, para. 3).**

In their response to comment 5, I find the figure for HCU and mortality rates for each income quintile over time to be very interesting, referred to as Figure S2 by the authors but appearing as S1 in the document.

**-Thanks for pointing out. S1 Fig. should be correctly labelled and referred to in the manuscript.**

For inpatient care and deaths, there seems to be an increasing gap between Q1 and Q2-Q5 rather than a broader increase in income-related inequality. I believe it is worth reflecting on this in the discussion. Pointing out that results are driven by the poorest group falling behind would help the reader correctly interpret the Q1/Q5 results. I recognise that the sole focus on lowest and highest group is stated in the abstract and introduction, however the title promises income-based differences in the Swedish population, the majority of which are in Q2-Q4.

**-We appreciate this suggestion and have now added this to the discussion (p.14, para. 4). Overall, however, when studying fig S1 of all 5 income quintiles separately, there seem to be rather robust and regular pattern of a parametric or “dose-dependent” relationship between income and most outcomes. However, as you pointed out, the scope and stated aim of our study is to describe Q1 vs Q5, why we focus on these rather than on other income groups.**

I would like to thank the authors for clarifying the estimates in response to comment 6, I now understand that the trend is a coefficient and thus on a different scale than the ORs.
Responding to comment 7, I believe FIgure 4 is now clearer and more consistent with the rest of the paper.
The comments 8-11 are adequately responded to.

**- Thanks for the feedback, we are glad to hear this.**


Reviewer #3: I think the authors have adequately addressed the questions/concerns raised by me and by the other reviewers. I think the paper is acceptable in its current form. Congratulations to the authors.

**-Thanks so much, and thanks for the valuable suggestions in the first round!**
