# Supplementary material for: Income-based differences in healthcare utilization in relation to mortality in the Swedish population between 2004–2017: A nationwide register study
Source: PLoS Med. 2023 Nov 16;20(11):e1004230. doi: 10.1371/journal.pmed.1004230 (PMC10653442; doi:10.1371/journal.pmed.1004230)
Supplement: Attachment — Submitted filename: reply_to_editor_3.docx [file pmed.1004230.s012.docx]

Dear Editor,

We are pleased to hear you are considering accepting our manuscript for publication. Thanks for suggestions and your feedback. Please see our responses, highlighted in **bold**, below.

-----

TITLE

Please revise the title to read, 'Income-based differences in health care utilization in relation to mortality in the Swedish population between 2004-2017: An observational cohort study.'

- **Thanks for the suggestion to include description of the study design. We think this is an improvement, and think the following adjustment could render an even more informative title “Income-based differences in health care utilization in relation to mortality in the Swedish population between 2004-2017: A nationwide register-based study”, highlighting the comprehensiveness of the study, similar to what was done in for instance this Finnish paper in PLOS Medicine earlier this year:** <https://journals.plos.org/plosmedicine/article?id=10.1371/journal.pmed.1004072>

**However, if you believe “Income-based differences in health care utilization in relation to mortality in the Swedish population between 2004-2017: An observational cohort study“ suits PLOS Medicine better, please feel free to use that title.**

TABLES

Table 1 – we asked previously for inclusion of a table with more detailed demographic data. Thank you for your response. Apologies for the lack of clarity. The editorial team have discussed this point and agree that it is important for the reader to appreciate how the population studied is representative of the nation and how that may have changed over time.

- **Thanks for feedback in clarifying the Table 1. Perhaps there might be a misinterpretation of Table 1. Table 1 (top rows) describe the entire the *entire* Swedish adult population (individuals aged 16 and above), as described for instance in the Abstract and in Methods (p 6, para. 4). Only a minimal proportion was excluded due to missing sociodemographic data or non-positive income data (in 2004, this was 0.4% of the Swedish population 16 or older, in 2017 it was 2%) (as described in the Methods, [p.8 para.2]). For obvious reasons, we cannot compare the representativeness of our study sample with a sample also including these few additional individuals, given their missing data.**
- **Given that our analysis covers over 98% of the population concerning specialized care and mortality, the cohorts outlined in Table 1 (top) are not just "representative of the nation." They essentially encompass the entire nation, or one could say, they 'perfectly represent' the nation. So, when we in the first sentence in the Results section (p 10) describe “**we investigated 105.3 million person-years at risk”**, the number is from the first cell in the Table 1. We have now further clarified Table 1(first column) and pertaining legend (p.8).**

Considering that a lower proportion of people in Q1 were born in Sweden, we think this is important detail.

- **Income groups were stratified with regard to age and birth year (as described in Methods), not country of birth, so your observation is expected. If one wants to compare Q1 with the entire population, just look with ‘Tot.pop’ for that variable. In fact, the table was designed in a way to facilitate comparisons between Q1, Q5, and the total population.**

Please include an updated table including baseline demographic details – age (including range), ethnicity, income group, marital status, sex etc.

- **Given the study's design with *repeated* cohorts, we are not sure what is meant by “baseline” demographic details. If it is anything beyond the info in Table 1, could you please specify what ”baseline demographic details“ refers to, given this is a repeated cross sectional (not a longitudinal) study?**
- **Again, just to assure no misunderstandings: Table 1 describes the population denominators (not only the individuals utilizing care, they are listed in Table S3) that we used in the analyses of the outcomes “Outpatient care, inpatient care, and mortality” and “Primary
  care”, respectively. In other words, Table 1 (top rows, columns ‘Tot. pop’) characterise the entire (>98%) Swedish adult population. This is now clarified in the first column and in the legend.**
- **Regarding age-ranges of the study populations, it thus spanned 16 yrs to the maximal age among Swedes. This should be clear from the abstract, Methods (p6. para. 4), and for clarity this is now also added to the legend of Table 1 (p.8).**

Please ensure that percentages are quantified with numerators and denominators.

- **The denominators are already specified in the N column. The numerator is simply the product of denominator times the percentages. Typing out the absolute numbers again for the denominator and numerator (7 digits x 2, for 3 groups and 2 variables makes another 84 digits, per row), is seems redundant and hides the interesting, easily comparable percentages. So to increase readability, we would prefer to not write out this product (percentage * denominator) and the denominator again, in the table. Let us know if you disagree.**

This is a prerequisite to publication. We encourage you to refer to currently published articles on our website <https://eur01.safelinks.protection.outlook.com/?url=https%3A%2F%2Fjournals.plos.org%2Fplosmedicine%2F&data=05%7C01%7Cpar.flodin%40ki.se%7C117ebe07035a48b251c208dbc9178d08%7Cbff7eef1cf4b4f32be3da1dda043c05d%7C0%7C0%7C638324875220526703%7CUnknown%7CTWFpbGZsb3d8eyJWIjoiMC4wLjAwMDAiLCJQIjoiV2luMzIiLCJBTiI6Ik1haWwiLCJXVCI6Mn0%3D%7C3000%7C%7C%7C&sdata=LZsOHKZnsyaDB0FNfTloEOC2wcJliWHIowMXDPZkFmE%3D&reserved=0> for suitable examples.

**As suggested, we once again revisited the PLOS Medicine website to review tables of study characteristics in all cohort or register studies published in 2023, identifying three that resembles our study (i.e., repeated cohorts at multiple time pointes), that provide such tables (could have missed some).**

[**https://journals.plos.org/plosmedicine/article?id=10.1371/journal.pmed.1004202**](https://journals.plos.org/plosmedicine/article?id=10.1371/journal.pmed.1004202) **lacks year-specific demographics, which we want to have.**

[**https://journals.plos.org/plosmedicine/article?id=10.1371/journal.pmed.1004072#**](https://journals.plos.org/plosmedicine/article?id=10.1371/journal.pmed.1004072) **mirrors our Table 1's demographic information, although we don’t show sex since we cover the entire adult population.**

[**https://journals.plos.org/plosmedicine/article?id=10.1371/journal.pmed.1004164#**](https://journals.plos.org/plosmedicine/article?id=10.1371/journal.pmed.1004164) **provides demographics only at study onset and not stratified by exposure groups, which we want to have.**

**We did not find any studies that described population characteristic in a way that better capture our very comprehensive, nationwide cohorts across multiple timepoints. We've enhanced the clarity of Table 1 by changing the row names in the first column and adding explanation in the legends. We are confident that Table 1 adequality characterize the study populations. If we have misinterpreted your feedback or if you disagree with our rationale, please let us know.**
